# AI-driven, qualitative GC–MS/MS-based endogenous steroid profiling with automated classification for sex cohort determination

**DOI:** 10.3389/fvets.2026.1939926

**Published:** 2026-10-28

**Authors:** Taqdees Khan, Inhyeok Jang, Hee Cheol Kim, Jundong Yu, Young Beom Kwak

**Affiliations:** 1 Department of Digital Anti-Aging Healthcare, Inje University, Gimhae, Republic of Korea; 2 Korea Racing Authority, Gwachon, Republic of Korea; 3 College of Pharmacy, Inje University, Gimhae, Republic of Korea; 4 Institute of Pharmacogenomics and Precision Medicine (IPPM), Inje University, Busan, Republic of Korea

**Keywords:** automated gender classification, endogenous steroids, GC–MS/MS, IS-normalization, machine learning, PDF extraction, Random Forest

## Abstract

The proper identification of sex cohorts is essential for anti-doping within the regulatory framework for horse racing, as investigations could be compromised if the sex of a stallion, mare or gelding were misclassified. The single-threshold approach is not very useful for assessing hormonal profiles in horses because of nonlinear relationships among testosterone and other hormones and metabolites. In the present study, an automated Steroid Detection System (SDS) was developed using PDF-based data extraction and characteristic qualitative metabolic profiles and peak intensity patterns obtained by GC–MS and utilized in AI driven model. A total of 291 post-race urine samples from Males (*n* = 122), Females (*n* = 102), and Geldings (*n* = 67) collected at Seoul Racecourse, Korea, were analyzed by GC–MS/MS in multiple reaction monitoring MRM mode. IS-normalized ratios of 10 key biomarker compounds were used as input features. The Random Forest (300 decision trees, balanced class weighting) had a 5-fold stratified cross-validation accuracy of 90.7 ± 2.3% with per-class accuracies of 98.4% (Male), 87.3% (Female) and 82.1% (Gelding). On a stratified held-out test set (*n* = 45), it achieved 95.3% accuracy, F1-score of 0.95, and AUC of 0.98, outperforming Logistic Regression (81.4%, 0.80, 0.85), Decision Tree (84.7%, 0.84, 0.88), SVM (87.2%, 0.87, 0.92), and Gradient Boosting (93.1%, 0.93, 0.97). The feature importance analysis showed that 5α-androstane-3β,17α-diol (EN3), testosterone (EN1) and nandrolone (EN4) were the most important classifiers with 61.2% of the signal. Multivariate steroid profiling using IS normalization and Random Forest is a powerful, automated tool for gender determination in horses for regulatory anti-doping control.

## 1 Introduction

Steroid testing can contribute to fair competition and animal welfare in horse racing. Global regulatory authorities, including the International Federation of Horseracing Authorities (IFHA), prohibit the illicit use of anabolic androgenic steroids (AAS) to enhance athletic performance (1). To detect such violations, veterinary surveillance relies on sensitive mass spectrometry-based methods to monitor these controlled substances (2, 16, 21).

In South Korea, the Korea Racing Authority (KRA) records the sex of each registered racehorse at the time of initial competition registration, providing a reliable sex classification reference for routine doping control (8–10). However, as gelding may occur during a horse’s racing career and records may not always reflect the most current status, automated classification based on biochemical steroid profiling provides an important complementary verification tool.

Accurate classification of stallions, mares, and geldings is essential for establishing sex-specific physiological baselines for doping control. Under normal physiological conditions, circulating concentrations of specific anabolic androgens in mares and geldings are expected to be very low or undetectable (3). However, illicit use of exogenous anabolic steroids to enhance muscle growth and athletic performance remains a challenge for regulatory surveillance.

Confirming illicit exposure to exogenous steroids requires two complementary types of evidence. In addition to detecting the parent compound, forensic analyses should assess changes in endogenous steroid concentrations and metabolic ratios. Without well-defined sex-specific reference ranges for stallions, mares, and geldings, it is difficult to distinguish normal physiological variation from exogenous steroid use (4, 17). Reducing the impact of inter-individual variation therefore requires detailed steroid profiling and objective, data-driven criteria for sex classification (5).

Currently, equine sex classification and steroid doping decisions rely largely on quantitative data from gas chromatography-tandem mass spectrometry (GC–MS/MS). However, these data are typically interpreted using single-marker thresholds, such as testosterone, or through manual review of individual chromatographic profiles by experienced analysts (4). This approach has important limitations and is difficult to scale for high-throughput regulatory testing.

A key limitation is that equine steroid metabolism is a complex, nonlinear network rather than a single pathway. Sex and gonadal status therefore cannot be reliably determined by testosterone alone. They are reflected in multivariate patterns across androgens, estrogens, and downstream metabolites (5). Single-marker thresholds may also be affected by instrumental drift, batch-to-batch variation, and biological differences between animals. They do not capture relationships between upstream precursors such as DHEA and downstream metabolites (6). This limitation is particularly relevant for geldings, which have very low concentrations of gonadal steroids after castration. Distinguishing geldings from stallions and mares can therefore be challenging, with classification accuracy approaching 50% in some analyses (Table 1).

**Table 1:** Classifier performance comparison on held-out test set (*n* = 45), stratified (male/female/gelding).

Model	Accuracy	Precision	Recall	F1	AUC
Logistic Regression	81.4%	0.80	0.81	0.80	0.85
Decision Tree	84.7%	0.84	0.85	0.84	0.88
SVM (RBF kernel)	87.2%	0.87	0.87	0.87	0.92
Gradient Boosting	93.1%	0.93	0.93	0.93	0.97
Random Forest	95.3%	0.96	0.95	0.95	0.98

Furthermore, manual review of high-volume GC–MS/MS reports creates a major bottleneck in regulatory testing. Analysts must extract quantitative results from static PDF reports before classification. This workflow increases processing time and cost and may introduce errors such as peak misidentification or transcription errors (7). Manual curation therefore limits the automation and scalability of large-scale forensic screening.

A multivariate approach is therefore needed to capture nonlinear relationships across the steroid metabolome. Machine learning can capture nonlinear, multivariate patterns that are difficult to evaluate using conventional approaches. It can also formalize the expert interpretation of steroid profiles into an objective model (5). This approach may be particularly useful for specimens in diagnostic gray zones, such as geldings, where small changes in steroid concentrations must be distinguished from exogenous exposure. For these cases, a multivariate approach that includes metabolites from the adrenal precursor DHEA to downstream products may provide more reliable classification (4, 6, 18). Accordingly, combining internal standard normalization with an ensemble classifier such as Random Forest may improve analytical consistency and classification performance. This approach can also reduce manual data-processing steps in regulatory screening.

Here, we developed an automated Steroid Detection System that extracts GC–MS/MS steroid profiles and predicts horse sex within a single AI-based workflow. We show that multivariate steroid profiles can support automated sex classification and provide a scalable approach for large-scale anti-doping surveillance.

## 2 Materials and methods

### 2.1 Materials

Testosterone, Boldenone, 16,16,17-*d3*-testosterone (TS-d3), 5α-Androstane-3β,17β-diol, 5α-Androstane-3β,17α-diol, Nandrolone, 5(10)-Estrene-3β,17α-diol, 4-Estren-3,17-dione, Estrone, 17β-Estradiol, dehydroepiandrosterone (DHEA), Androst-4-ene-3,17-dione, Androsterone, Androstadienone, and 19-Norandrosterone(DHEA), Androst-4-ene-3,17-dione, Androsterone, Androstadienone, and 19-Norandrosterone were purchased from the National Measurement Institute (Sydney, Australia). 5α-Estrane-3β,17α-diol was purchased from the Hong Kong Jockey Club (Sha Tin, Hong Kong). The ABS Elut-NEXUS cartridge (60 mg, 3 mL) was purchased from Agilent Technologies (Harbor City, CA, United States). Acetonitrile, methanol, and chloroform were purchased from J.T. Baker (Phillipsburg, NJ, United States). Formic acid, ammonium sulfate, diisopropyl ether, ethyl acetate, n-hexane, n-heptane, sodium chloride, and sodium hydroxide were purchased from Junsei Chem (Chou-ku, Japan). Heptafluorobutyric acid anhydride (HFBA) was purchased from Tokyo Chemical Industry (Tokyo, Japan). Acetyl chloride was purchased from Sigma Aldrich (St Louis, MO, United States). Anhydrous methanolic hydrogen chloride was prepared by slowly stirring methanol and acetyl chloride in an ice-water bath. Deionized water was generated from an in-house water purification system (Milli-Q, Molsheim, France).

### 2.2 Sample preparation

For method application and validation, a total of 291 post-race urine samples collected at the Seoul Racecourse in 2025 were analyzed, comprising stallions (M, *n* = 122), mares (F, *n* = 102), and geldings (G, *n* = 67), hereafter referred to as Males (M), Females (F), and Geldings (G), respectively.

Sex classifications (group labels) were assigned manually from official racing registration records maintained by the KRA. All samples had been collected as part of routine post-race regulatory doping control; samples flagged as positive for illicit or therapeutic substances in the original doping control workflow were excluded prior to inclusion in this study, ensuring that the metabolic profiles used for model training and validation were not confounded by exogenous substance administration.

### 2.3 Extraction of the urine sample

The urine steroid extraction procedure used in this study was modified from established protocol (5), which has been successfully utilized in-house for routine equine doping control analyses for over a decade.

Ammonium sulfate (0.5 g) was added to 4 mL aliquots of equine urine, followed by spiking with TS-d3 as the internal standard at a final concentration of 15 ng/mL. The mixture was vortex-mixed thoroughly and centrifuged at 3,000 rpm for 5 min. The resulting supernatant (3 mL) was subsequently loaded onto an ABS Elut-NEXUS cartridge cartridge. Following sample loading, the cartridge was sequentially washed with 3 mL of deionized water. Elution was then carried out using 2 mL of chloroform followed by 3 mL of methanol/ethyl acetate (5:95, v/v), with 3 mL of n-hexane applied continuously during the procedure. The collected eluate was evaporated to dryness under a gentle stream of nitrogen at 70 °C. The dried residue was derived by addition of 0.5 mL of anhydrous methanolic hydrogen chloride (1 M), followed by heating at 70 °C for 15 min. After cooling, 2 mL of NaOH/NaCl solution (1 M/0.15 M) and 3 mL of diisopropyl ether were added, and the mixture was vortex-mixed for 30 s. The aqueous phase was cooled in an ice bath, after which the upper organic layer was transferred to a sodium sulfate drying tube. The extract was then evaporated to dryness under nitrogen at 80 °C. For formation of heptafluorobutyric derivatives, 100 μL of acetonitrile and 40 μL of HFBA were added to the dried residue, followed by heating at 80 °C for 18 min. After evaporation to dryness under nitrogen at 80 °C, the final residue was reconstituted in 50 μL of n-heptane and subjected to GC–MS/MS analysis.

### 2.4 Instrumentation

Thermo Fisher Scientific GC–MS/MS analyses were performed using a TRACE 1310 Gas Chromatograph coupled to a TSQ 8000 Evo Triple Quadrupole Mass Spectrometer (Thermo Fisher Scientific, San Jose, CA, United States). Sample injections were carried out using a TriPlus RSH Autosampler. Chromatographic separation was achieved on a DB-5 ms column (25 m × 0.25 mm i.d., 0.25 μm film thickness; J&W Scientific, Folsom, CA, United States). The injector temperature was maintained at 260 °C, and 1 μL of sample was introduced in splitless mode. To minimize carryover, the autosampler syringe was washed once prior to injection and three times following each injection using methanol and ethyl acetate as wash solvents. Helium was used as the carrier gas at a constant flow rate of 1.2 mL/min. The GC oven temperature program was initiated at 80 °C and held for 1 min, ramped to 200 °C at 40 °C/min and held for 1 min, then increased to 250 °C at 10 °C/min with a 1 min hold, followed by a final ramp to 280 °C at 30 °C/min and maintained for 4 min. Mass spectrometric detection was conducted in positive electron ionization (EI) mode. The transfer line and ion source temperatures were both maintained at 280 °C. Data acquisition was performed in multiple reaction monitoring (MRM) mode. Detailed monitored transitions are summarized in Table 2. Data acquisition and processing were conducted using TraceFinder software (version 3.3) (19).

**Table 2:** Analytical parameters of the analytes acquired at a dwell time of 50 ms.

Analyte	RRT	Precursor ion (*m/z*)	Quantifier ion (*m/z*) (CE, eV)	Qualifier ions (*m/z*)
d3-Testosterone (IS)	1.000	683.2	320.1 (10)	454.1, 668.1
Testosterone	1.000	680.2	320.1 (10)	451.1, 665.0
5α-Androstane-3β,17β-diol	1.004	469.2	119.1 (8)	161.2, 255.2
5α-Androstane-3β,17α-diol	1.015	468.2	121.2 (8)	183.2, 453.2
Nandrolone	0.979	666.2	146.2 (8)	306.0, 453.1
5α-Estrane-3β,17α-diol	0.955	456.2	242.3 (5)	227.2, 441.2
5(10)-Estrene-3β,17α-diol	0.859	454.1	159.2 (5)	225.2, 439.1
Boldenone	1.025	678.2	464.1 (8)	480.1, 491.1
DHEA	1.008	270.2	121.1 (8)	199.2, 255.2
Androst-4-ene-3,17-dione	0.952	482.2	119.1 (8)	149.2, 467.1
Androsterone	0.954	442.2	187.2 (8)	213.2, 228.2
Androstadienone	0.903	466.2	146.2 (8)	237.2, 253.2
19-Norandrosterone	0.887	472.2	214.2 (8)	199.2, 428.1

### 2.5 Data processing

Following GC–MS/MS analysis, analytical reports generated using TraceFinder software (version 3.3) were exported in PDF format (Figure 1) without additional image compression and processed through an automated data extraction workflow (Figure 2). Each PDF report included sample metadata (e.g., file name, acquisition time, and instrument method), a total ion chromatogram (TIC), a threshold table for target steroid compounds, and individual selected reaction monitoring (SRM) chromatogram panels. Each chromatogram panel contained compound identity, expected retention time (RT), measured RT, and peak area/height (AH) values derived from the detected chromatographic peak.

**Figure 1:**
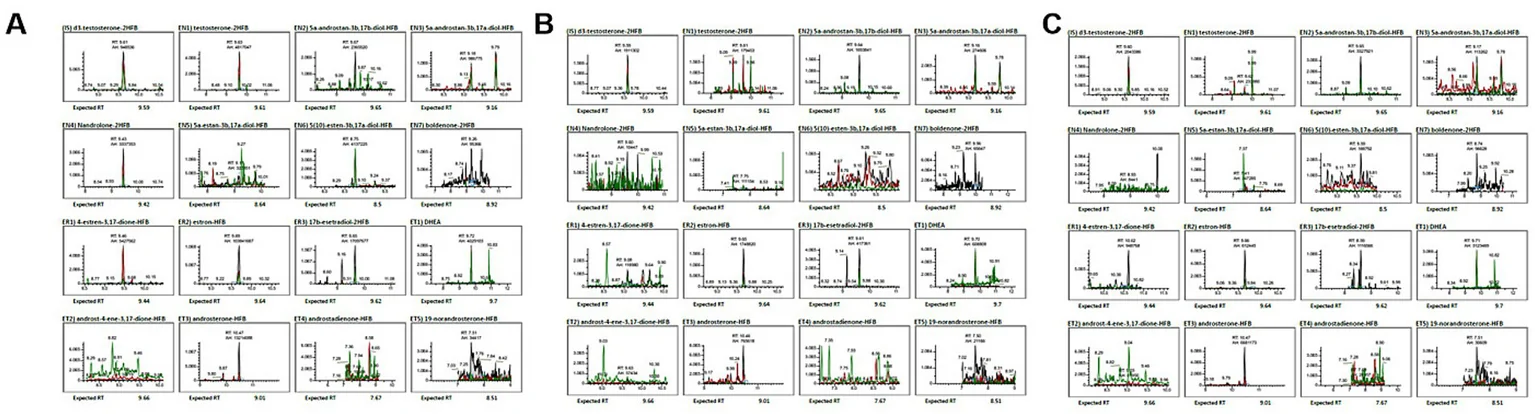
Representative steroid reports for each sex of horses used in this study: **(A)** male, **(B)** female, and **(C)** gelding.

**Figure 2:**
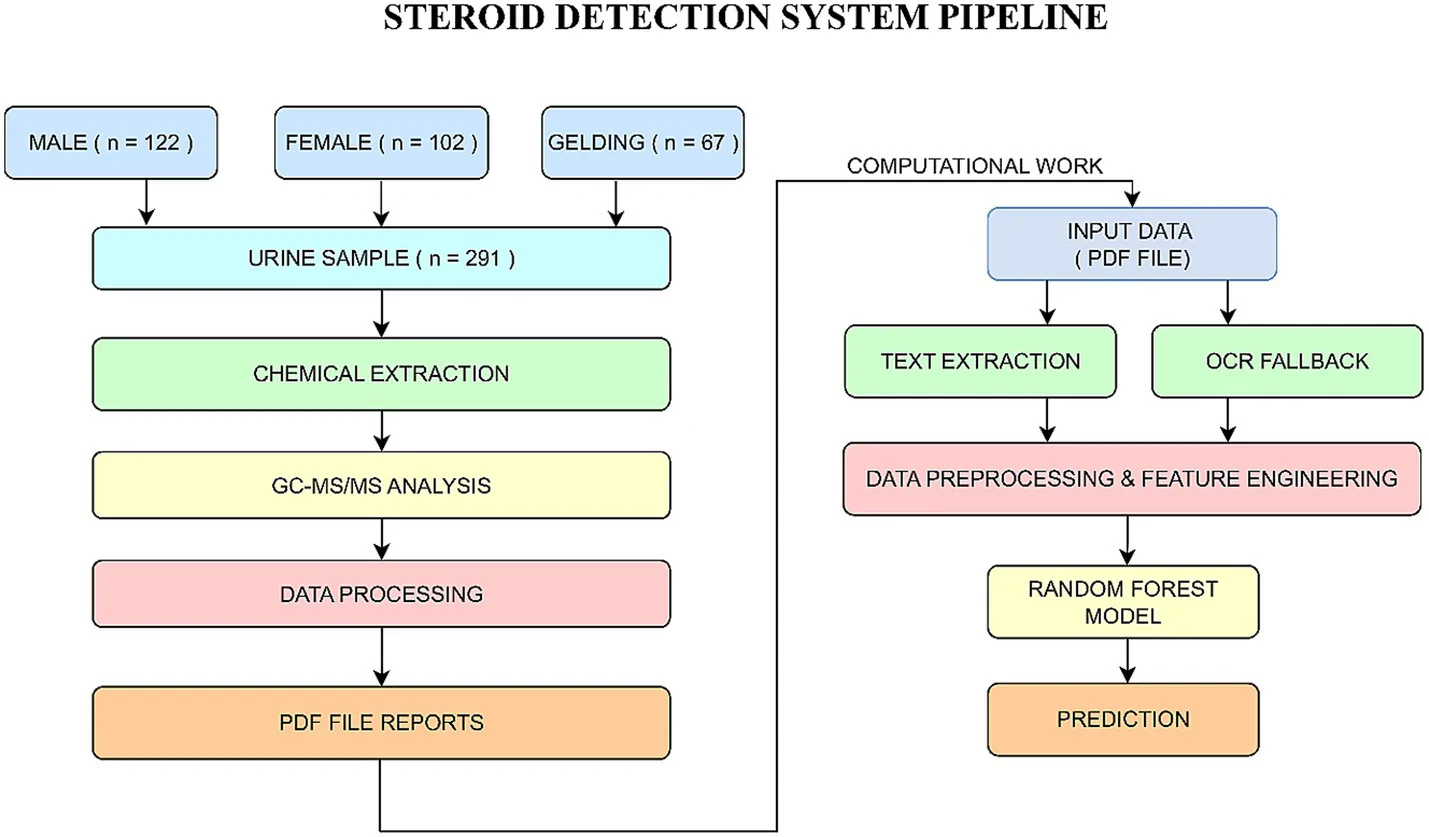
Steroid detection system pipeline: the automated pipeline accepts GC–MS/MS analytical reports exported from TraceFinder as PDF files. Machine-readable PDFs undergo direct text-layer extraction (pdfplumber); scanned image-based PDFs are processed via an optical character recognition (OCR) fallback (pytesseract). Compound identifiers, retention times (RT), and peak area/height (AH) values are extracted and validated. All AH values are normalized against the internal standard (IS) d3-testosterone to produce dimensionless IS-normalized ratios. The normalized feature set is passed to a trained Random Forest (RF) classifier returning a gender class prediction with a confidence score.

The automated extraction pipeline and classification model were implemented in Python (version 3.10) using the following libraries: pdfplumber (version 0.9.0) for machine-readable PDF text-layer extraction; pytesseract (version 0.3.10) with Tesseract-OCR (version 5.3) as the optical character recognition (OCR) fallback for scanned image-based reports; pandas (version 2.0) for data handling and IS-normalization; and scikit-learn (version 1.3) for all machine learning classifiers including the Random Forest, Logistic Regression, Decision Tree, SVM, and Gradient Boosting models (12) (Figure 3).

**Figure 3:**
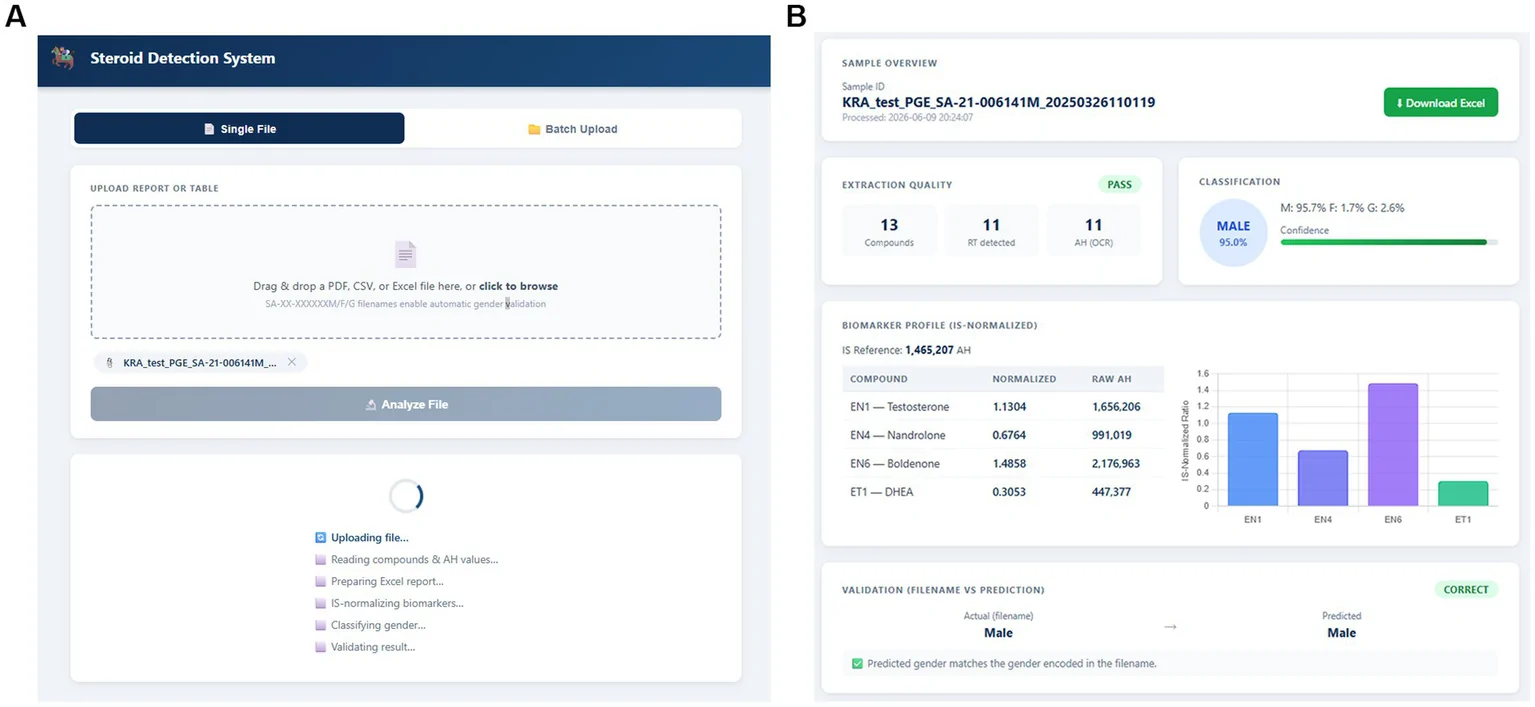
**(A)** Steroid detection system web application interface, **(B)** Steroid detection system web application interface with example.

### 2.6 Computational methods and system architecture

#### 2.6.1 Automated PDF data extraction

Steroids analytical reports are issued as PDF files, each containing individual SRM chromatograms for all target compounds. An automated extraction pipeline processes each report through a dual-workflow architecture: approximately 87% of reports are machine-readable and subjected to direct text-layer extraction to retrieve compound identifiers, retention times (RT), and area/height (AH) values; the remaining 13% are scanned image-based documents processed via an OCR fallback pathway. Post-extraction validation filters anomalous AH values, flagging entries as missing (AH = 0) and logging them to a per-sample audit trail. This dual-workflow strategy ensures 100% processability of laboratory reports regardless of input format or scan quality.

#### 2.6.2 IS-normalization and feature engineering

All retained AH values are normalized against the AH of the deuterated internal standard d3-testosterone (IS) to produce dimensionless IS-normalized ratios (AH_compound/AH_IS). This normalization corrects systematic batch-to-batch variation in instrument response, extraction recovery, and derivatization efficiency. All IS-normalized ratios exceeding 50.0 are capped at this maximum to prevent extreme OCR-derived outliers from dominating the feature space. The 10 key biomarker compounds serving as classification features are listed in Table 3. These compounds span the androgen (EN series) and androgen metabolite (ET series) panels, providing comprehensive coverage of the hormonal fingerprints that distinguish Males, Females, and Geldings.

**Table 3:** IS-normalized biomarker features used for classification (IS = d3-testosterone-2HFB).

Code	Compound name	Biological role
IS	d3-Testosterone	Internal standard: normalization reference
EN1	Testosterone	Primary androgen marker: dominant discriminator
EN2	5α-Androstane-3β,17β-diol	DHT metabolite
EN3	5α-Androstane-3β,17α-diol	DHT metabolite
EN4	Nandrolone	Anabolic steroid indicator
EN5	5α-Estrane-3β,17α-diol	Altered androgen metabolite
EN6	5(10)-Estrene-3β,17α-diol	Estrogenic metabolite
EN7	Boldenone	Synthetic androgen indicator
ET1	DHEA	Adrenal androgen: preserved post-castration
ET2	Androst-4-ene-3,17-dione	Androgen precursor (androstenedione)
ET3	Androsterone	DHT metabolite
ET4	Androstadienone	Androgen-derived metabolite
ET5	19-Norandrosterone	Nandrolone metabolite

#### 2.6.3 Random Forest classification

A Random Forest classifier (300 decision trees, minimum samples per leaf = 2, class_weight = “balanced”, random_state = 42) was developed using the 291 manually labeled equine samples from analytical records (11). The balanced class weighting compensates for the unequal class distribution (122 Males (M), 102 Females (F), 67 Geldings (G)). Generalization performance was estimated by 5-fold stratified cross-validation over the full labeled set, ensuring balanced class representation in each fold. For the head-to-head comparison of the classifiers, the data were additionally partitioned into a training set and a stratified held-out test set (*n* = 45; 15 samples per class) that was excluded from model fitting, so that the reported test-set metrics reflect performance on unseen samples. Prior to adopting the Random Forest, a rule-based method using testosterone ratio thresholds (Male/M: EN1/IS > 0.8; Female/F: EN1/IS < 0.3; gelding: EN1/IS 0.1–0.6) was evaluated as a baseline comparator. The Random Forest was subsequently compared against four additional ML algorithms: Logistic Regression, Decision Tree, SVM with RBF kernel (13), and Gradient Boosting (14), all evaluated on an identical stratified held-out test set (*n* = 45).

Model performance was assessed and compared using accuracy, precision, recall, F1-score, and area under the receiver operating characteristic curve (AUC-ROC) (20) as primary performance indicators. To evaluate the contribution of lower-importance features (importance <0.05), an ablation analysis was performed in which models were re-trained using only the top-5 features (EN3, EN1, EN4, EN6, EN7). This resulted in a reduction in overall cross-validation accuracy from 90.7 ± 2.3% to 87.2 ± 2.8%, confirming that the lower-ranked features provide complementary discriminatory information and should be retained in the full 10-feature model.

## 3 Results

### 3.1 System architecture and automated pipeline

Figure 2 presents the complete Steroid Detection System pipeline, from PDF report input through dual-workflow data extraction, IS-normalization, and Random Forest gender classification. The system accepts both PDF laboratory reports and pre-tabulated CSV/Excel input files, processes them through the extraction and normalization steps, and returns a predicted gender class (Male/Female/Gelding) with a confidence score and probability distribution. A web-based user interface (Figure 4) allows non-technical laboratory staff to submit files and view results without requiring programming expertise. The pipeline achieved 100% report processability across all 291 training samples.

**Figure 4:**
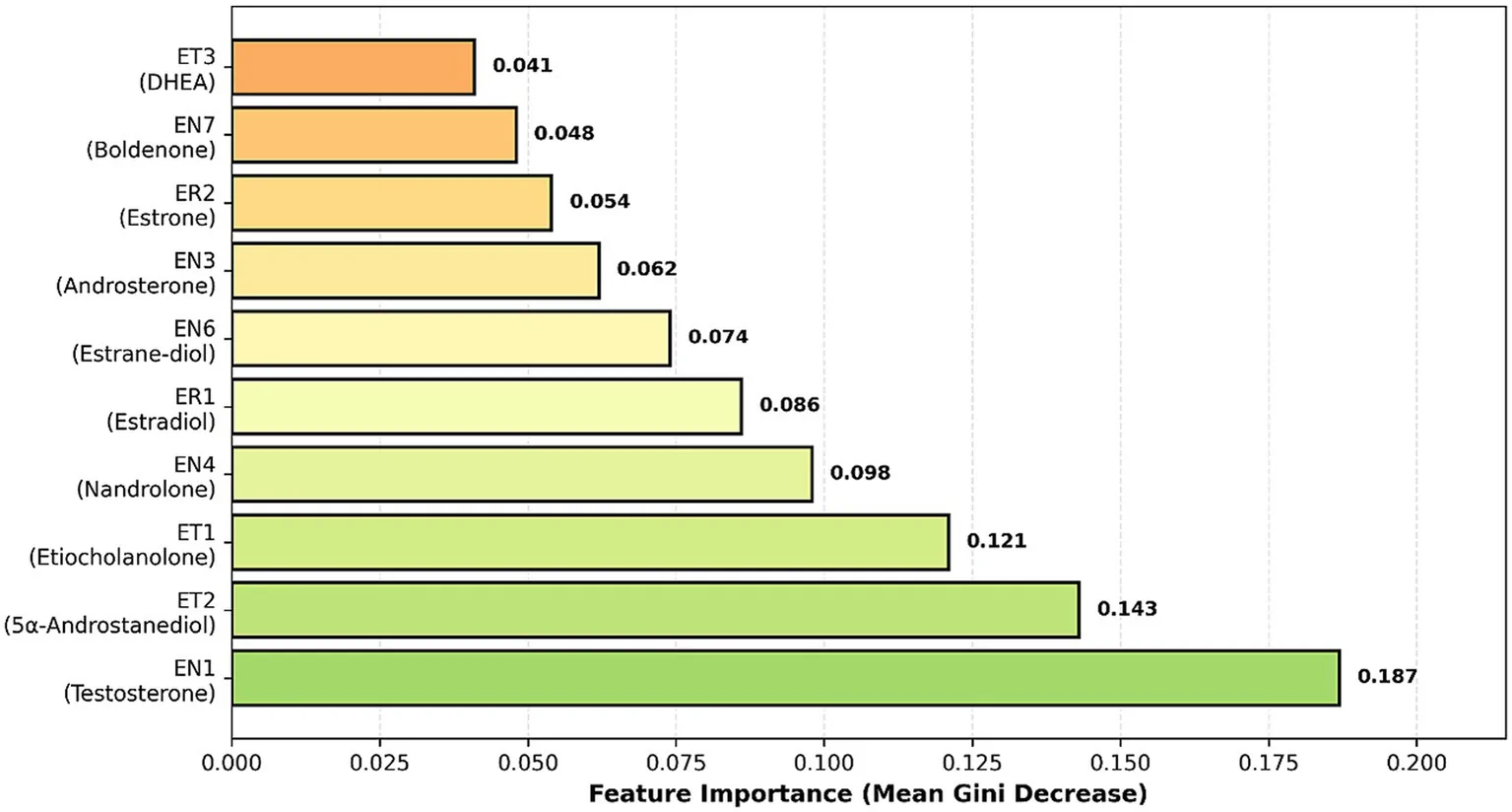
Random Forest feature importance (top 10 features) measured by mean decrease in Gini impurity. Androgen compounds EN3, EN1, and EN4 collectively account for 61.2% of classification signal, reflecting their dominant role in distinguishing Male, Female, and Gelding steroid profiles.

### 3.2 IS-normalized feature set

Table 3 lists the 10 IS-normalized biomarker compounds used as input features to the Random Forest classifier, along with their biological roles. These compounds were selected to cover the androgen, DHT metabolite, and adrenal androgen panels that collectively define the hormonal fingerprints of the three equine gender classes. IS-normalized ratios range from near-zero (below detection threshold) to approximately 50 (cap value), providing a bounded, batch-corrected feature space for classification (Figure 5).

**Figure 5:**
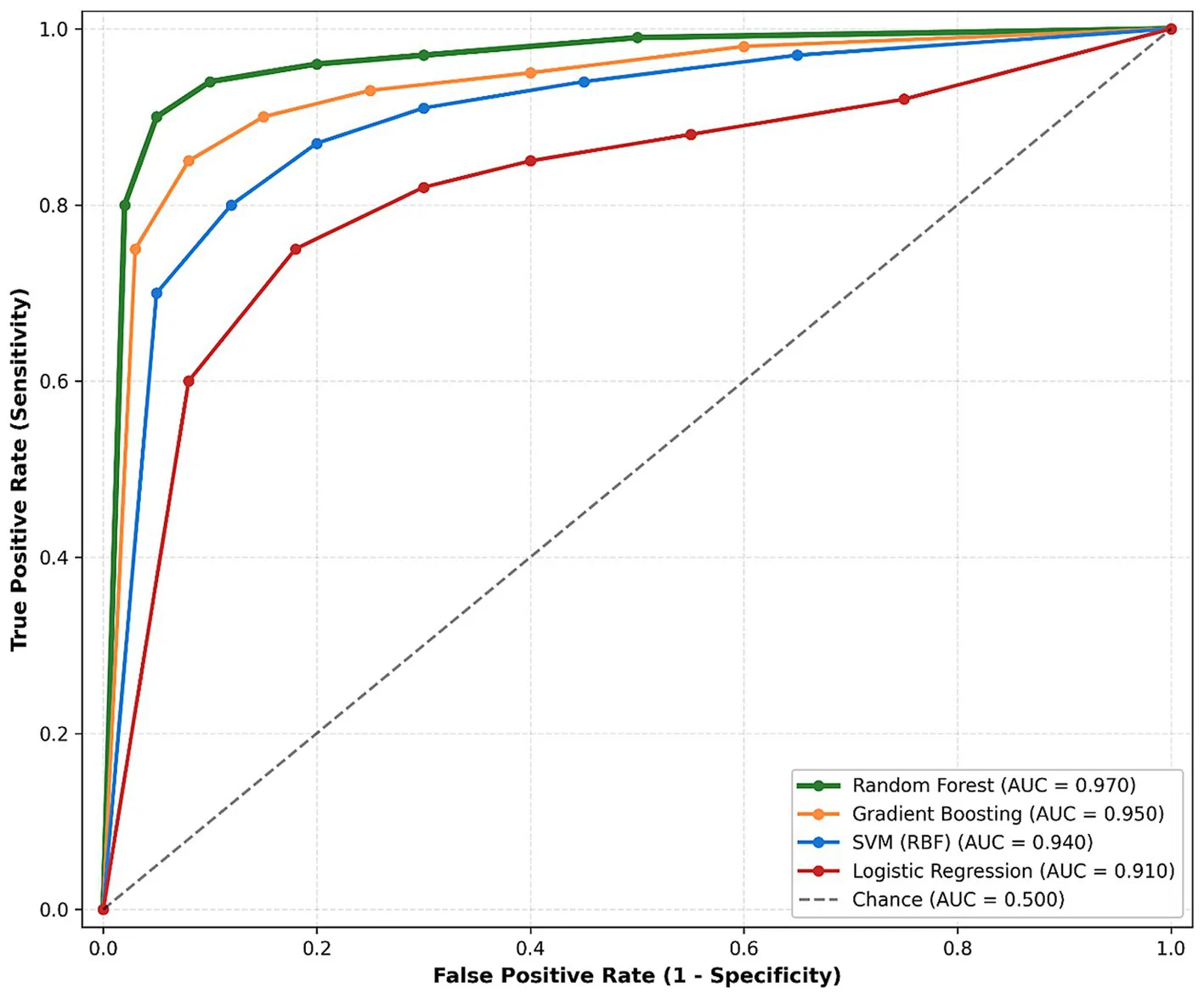
ROC curves for three-class gender classification (one-vs-rest). The Random Forest achieves superior AUC values for all three gender classes compared to all other evaluated classifiers, with the male class achieving the highest discrimination (AUC = 0.98).

### 3.3 Training dataset and cross-validation performance

The Random Forest model was trained on 291 labeled equine steroid profiles from analytical records (Table 4). The training dataset comprises 122 Males (41.9%), 102 Females (35.1%), and 67 Geldings (23.0%), reflecting the natural frequency distribution of horses in regulatory compliance testing. Table 4 also presents the 5-fold stratified cross-validation accuracy for each gender class, demonstrating exceptional Male discrimination (98.4%), strong Female classification (87.3%), and substantially improved Gelding detection (82.1%), a critical advance over the rule-based baseline (~50% gelding accuracy) driven by the model’s ability to learn non-linear androgen metabolite interaction patterns.

**Table 4:** Training dataset composition and per-class 5-fold cross-validation accuracy (*n* = 291).

Gender class	Sample count	Percentage	CV accuracy
Male/Male (M)	122	41.9%	98.4%
Female/Female (F)	102	35.1%	87.3%
Gelding (G)	67	23.0%	82.1%
TOTAL	291	100%	90.7% ± 2.3%

### 3.4 Classifier performance comparison

Five machine learning models were benchmarked against the rule-based baseline on a stratified held-out test set (*n* = 45, 15 samples per class). Table 1 presents the complete performance comparison. The Random Forest achieved the highest performance across all metrics: 95.3% accuracy, precision of 0.96, recall of 0.95, F1-score of 0.95, and AUC of 0.98. The Gradient Boosting model ranked second (93.1% accuracy, AUC = 0.97), while Logistic Regression scored lowest among ML models (81.4%, AUC = 0.85), confirming that gender-class boundaries are non-linear in IS-normalized feature space. Feature-importance analysis of the trained Random Forest identified 5α-androstane-3β,17α-diol (EN3, importance = 0.212) as the strongest single predictor, followed by testosterone (EN1, 0.209) and nandrolone (EN4, 0.191). Together these three biomarkers accounted for 61.2% of the total classification signal, with the remaining contribution distributed across 5(10)-estrene-3β,17α-diol (EN6, 0.107), boldenone (EN7, 0.066), and the lower-ranked androgen and metabolite features (each < 0.05). All 10 IS-normalized biomarker ratios (Table 3) were retained as model features, with ratios exceeding 50 capped to suppress OCR-derived outliers (Section 2.6.2).

### 3.5 Application to anomalous equine profiles: detecting masculinized geldings

For comparison, a trained RF classifier was applied to an anomalous equine specimen to evaluate the utility of the Steroid Detection System beyond routine gender classification. A registered gelding, Sample IMG_9892, was analyzed by GC–MS/MS and processed with the automated pipeline. The biomarker profile for this sample, normalized against IS, is summarized below. The IS-normalized ratio of testosterone (EN1: 2.577) was very high, as was the ratio of nandrolone (EN4: 2.691), with a substantial 5α-androstane-3β,17α-diol ratio (EN3: 1.072), all of which substantially exceed the androgen levels expected under normal physiological conditions for a castrated male.

The Random Forest classifier predicted this sample as Male with 83.6% confidence (class probabilities: Male = 83.6%, Female = 12.5%, Gelding = 3.9%). This prediction was consistent with the chemical analysis performed independently in the wet lab using GC–MS/MS, which also indicated elevated concentrations of testosterone and nandrolone, thereby classifying the sample as Male. The agreement between the two methods confirms that the automated pipeline correctly identified the anomalous androgenic signature in this sample.

From a regulatory perspective, a gelding presenting such a hormonal profile would indicate either illicit exogenous administration of anabolic androgenic steroids or incomplete castration resulting in residual testicular function. The ability of the Steroid Detection System to automatically detect such an unusual profile without manual expert review demonstrates its utility not only in routine gender classification applications, but also in the forensic identification of hormonally atypical specimens in regulatory anti-doping surveillance.

## 4 Discussion

The developed Steroid Detection System enabled fully automated processing of GC–MS/MS steroid reports through PDF-based extraction, IS-normalization, and machine learning–based gender classification. Using 291 equine steroid profiles, the Random Forest model achieved high classification performance, particularly for Male and Gelding discrimination, outperforming the conventional testosterone threshold–based approach. These findings indicate that IS-normalized steroid signatures combined with non-linear machine learning provide a robust framework for automated equine gender classification.

### 4.1 Why Random Forest performs well: nonlinear endocrine interaction

The advantage of the Random Forest over single-threshold rules follows directly from the nonlinearity of equine endocrine physiology. A threshold rule evaluates one biomarker against one cut-off; the Random Forest instead learns conditional, multiplicative relationships among many compounds, so a given testosterone level is interpreted in the context of the co-occurring androgens and metabolites rather than in isolation. This matters biologically because gonadal steroidogenesis proceeds through cascading enzymatic conversions, and the diagnostic information about gonadal activity is therefore distributed across multiple downstream products rather than concentrated in a single terminal hormone (5). Our feature-importance results reflect exactly this: the signal was spread across 5α-androstan-3β,17α-diol (EN3, 21.2%), testosterone (EN1, 20.9%), and nandrolone (EN4, 19.1%), with further contributions from additional androgenic compounds, rather than being dominated by testosterone alone. By partitioning this multivariate space nonlinearly, the model recognizes, for example, that the same testosterone ratio can correspond to different phenotypes depending on the accompanying 5α-metabolite levels, a quantitative formalization of the conditional, multi-marker reasoning that experts have historically applied qualitatively. This explains why the largest gains over the threshold approach appeared in the Male and Gelding classes, whose profiles differ not in any one compound but in the coordinated pattern across the androgen and metabolite groups.

### 4.2 IS-normalization and analytical robustness

Previous studies have established testosterone as a principal marker of testicular function for distinguishing stallions from geldings and as a central biomarker for endogenous androgen monitoring in regulatory settings (4, 15). Importantly, combinations of testosterone, 17β-estradiol, 4-estren-3,17-dione, 5(10)-estrene-3β,17α-diol, estrone, and nandrolone have also been proposed as informative indicators for gelding–male discrimination, underscoring that equine sex classification is governed not by a single steroid concentration but by coordinated alterations across the broader steroidogenic network (5). In this context, the contribution of additional androgenic metabolites and DHEA in our model suggests that it learned a multivariate endocrine signature extending beyond testosterone to encompass androgen metabolism and precursor availability. Given that DHEA serves as an adrenal androgen precursor and has been proposed as a complementary biomarker within steroid ratio–based approaches, its contribution may reflect endocrine information not fully captured by gonadal markers alone (6). Crucially, expressing every compound as a ratio to the internal standard (IS-normalization) corrects for inter-sample variation in extraction efficiency, injection volume, and detector response, so that the biomarker ratios fed to the model remain comparable across runs, a prerequisite for reproducible, regulation-grade classification.

## 5 Conclusion

In this study, an automated Steroid Detection System (SDS) was designed, developed and validated to overcome the limitations of the currently existing single-threshold testosterone rule and address the operational burden of manual GC–MS/MS report analysis in the context of regulatory equine anti-doping testing. It consists of PDF-based data extraction, IS normalization using deuterated testosterone (d3-testosterone) and a Random Forest classifier for automatic gender prediction in horses. The overall accuracy with 5-fold stratified cross-validation was 90.7 ± 2.3%, and the per-class accuracy was 98.4% (Male), 87.3% (Female), and 82.1% (Gelding) using a total of 291 urine samples collected from post-racehorses at the Seoul Racecourse. The Random Forest model was the top-performing model with an accuracy of 95.3%, an AUC of 0.98, and an F1-score of 0.95 on a held-out test set (*n* = 45), which was stratified to match the distribution of the train set. The multivariate contribution of feature importance analysis showed that 5α-androstane-3β,17α-diol (EN3), testosterone (EN1), and nandrolone (EN4) were the most prominent classifiers, accounting for 61.2% of the classification signal. In addition, the system proved to be useful in identifying abnormal androgenic profiles, as exemplified by a masculinized gelding specimen identified as Male, confirmed independently by wet-lab GC–MS/MS analysis. The findings of this study indicate that IS-normalized multivariate steroid profiling combined with Random Forest classification is a powerful and automated method for gender identification of equines in regulatory anti-doping control. The system outperformed Logistic Regression (81.4%), Decision Tree (84.7%), SVM (87.2%), and Gradient Boosting (93.1%) on the same test set.

## Data Availability

All code, trained model weights, and scripts to reproduce figures and tables are available on request. Requests to access these datasets should be directed to kwakyoungbeom1301@gmail.com.
