# Supplementary material for: AI-driven, qualitative GC–MS/MS-based endogenous steroid profiling with automated classification for sex cohort determination
Source: Front Vet Sci. 2026 Oct 28;13:1939926. doi: 10.3389/fvets.2026.1939926 (PMC13753903; doi:10.3389/fvets.2026.1939926)
Supplement: Supplementary file 1 [file Data_sheet_1.docx]

**Method Validation**

The proposed GC–MS/MS screening method for anabolic steroids in equine urine was validated with reference to the guidelines of the United Nations Office on Drugs and Crime (UNODC) and the EURACHEM guide. The validation parameters included selectivity, screening detection limit (SDL), and repeatability.

**Screening detection limit**

The screening detection limits SDL was determined according to the EURACHEM guidelines. A mixed standard solution was initially spiked into blank mare urine at a concentration of 50 ng/mL and subsequently diluted with blank urine to prepare five concentration levels (0.1, 1, 2, 5, and 10 ng/mL). Each concentration level underwent the complete sample preparation procedure and was analyzed in 10 replicate measurements performed on different days. The SDL was defined as the lowest concentration at which all target analytes were consistently detected in all 10 replicate measurements, with a relative standard deviation (RSD) of retention times (RTs) of less than 2%.

**Repeatability**

Repeatability was evaluated in accordance with the UNODC guidelines. Five replicate analyses (n = 5) of urine samples at a fixed concentration were performed within a single day under the same analytical conditions. Repeatability was assessed based on the false-negative rate and retention-time variability. The method was considered acceptable when the false-negative rate was below 20% and the RSD of RTs was less than 2%.

**Statistical Analysis**

Statistical analyses were conducted using Python (version 3.10.12) with the pandas and scipy libraries. To evaluate differences in the mean concentrations (peak areas) of targeted steroid metabolites among the three gender groups (M, F, and G), a one-way analysis of variance (ANOVA) was performed. For metabolites that exhibited statistically significant differences (*p* < 0.05), Tukey's Honestly Significant Difference (HSD) post-hoc test was applied to identify specific group-wise variations. Statistical significance levels were denoted as **p* < 0.05, ***p* < 0.01, and ****p* < 0.001.

**Results**

**Screening Detection Limits**

The SDLs of the target anabolic steroids in equine urine were determined by evaluating the lowest concentration at which each analyte was successfully detected in all ten replicate analyses (n = 5) with an RT variation. As summarized in Table S1, most of the target analytes, including testosterone, boldenone, DHEA, and various diol metabolites, exhibited an SDL of 0.5 ng/mL. Nandrolone and mibolerone showed SDLs of 1.0 ng/mL and 2.0 ng/mL, respectively.

**Repeatability**

The short-term precision (repeatability) of the analytical method was evaluated by performing five replicate analyses (n = 5) urine samples spiked at a constant concentration within a single day. The variations in RT across all groups showed a maximum standard deviation (SD) of 0.02 min, with relative RSDs ranging from 0.00% to 0.25%, demonstrating high analytical precision and reliability for routine screening.

**Comparison of Steroid Metabolite Profiles Across Gender Groups**

As this study focused on qualitative profiling rather than absolute quantification, the relative abundances of the compounds were evaluated using their mass spectrometry signal intensities (peak height). Accordingly, the intensity profiles of 13 targeted steroid metabolites were compared across the three gender groups Male (M), Female (F), and Gelding (G). Subsequent multiple comparison analysis using Tukey's HSD demonstrated that for all metabolites showing significant variance, the M group exhibited a distinct profile clearly differentiated from both the F and G groups. Notably, no statistically significant differences were observed between the F and G groups for any of the analyzed compounds. Based on the significance patterns, the metabolites were categorized into three distinct subsets. Overall, eight metabolites including testosterone, nandrolone, and their related derivatives demonstrated significantly higher mean concentrations in the M group compared to both the F and G cohorts (ranging from *p* < 0.05 to *p* < 0.001). In contrast, five metabolites, such as DHEA and boldenone, showed no significant gender-dependent variance (*p* > 0.05). Detailed statistical parameters, including group-wise means, variances, and specific post-hoc *p*-values for all evaluated compounds, are summarized in Table S2.

**Table S1. Screening detection limits (SDLs) and repeatability of the target analytes in equine urine.**

| **Target Analytes** | **SDL (ng/mL)** | **Repeatability** |
| --- | --- | --- |
| Testosterone | 0.5 | RT SD ≤0.02 min; RSD ≤0.25% |
| 5α-Androstane-3β,17β-diol | 0.5 | RT SD ≤0.02 min; RSD ≤0.25% |
| 5α-Androstane-3β,17α-diol | 0.5 | RT SD ≤0.02 min; RSD ≤0.25% |
| Nandrolone | 1.0 | RT SD ≤0.02 min; RSD ≤0.25% |
| 5α-Estrane-3β,17α-diol | 0.5 | RT SD ≤0.02 min; RSD ≤0.25% |
| 5(10)-Estrene-3β,17α-diol | 0.5 | RT SD ≤0.02 min; RSD ≤0.25% |
| Boldenone | 0.5 | RT SD ≤0.02 min; RSD ≤0.25% |
| DHEA | 0.5 | RT SD ≤0.02 min; RSD ≤0.25% |
| Androst-4-ene-3,17-dione | 0.5 | RT SD ≤0.02 min; RSD ≤0.25% |
| Androsterone | 0.5 | RT SD ≤0.02 min; RSD ≤0.25% |
| Androstadienone | 0.5 | RT SD ≤0.02 min; RSD ≤0.25% |
| 19-Norandrosterone | 0.5 | RT SD ≤0.02 min; RSD ≤0.25% |

Table S2. One-way ANOVA and Tukey's post-hoc analysis of metabolite profiling across gender groups.

| Compound | Male (M) | | Female (F) | | Gelding (G) | | F-statistic | p-value | M vs F | M vs G | F vs G |
| --- | --- | --- | --- | --- | --- | --- | --- | --- | --- | --- | --- |
|  | Mean | SD | Mean | SD | Mean | SD |  |  |  |  |  |
| 5(10)-Estrene-3β,17α-diol | 9,704,751 | 1.32 × 10¹⁴ | 239,230.8 | 2.64 × 10¹⁰ | 258,629.1 | 6.37 × 10¹⁰ | 32.93 | < 0.0001 | *** | *** | ns |
| 5α-Androstane-3β,17α-diol | 3,008,418 | 5.47 × 10¹² | 392,206.4 | 6.69 × 10¹¹ | 100,147.1 | 2.60 × 10⁹ | 99.95 | < 0.0001 | *** | *** | ns |
| 5α-Estrane-3β,17α-diol | 907,203.1 | 1.32 × 10¹² | 317,336.4 | 1.82 × 10¹¹ | 212,571.3 | 5.09 × 10¹⁰ | 18.69 | < 0.0001 | *** | *** | ns |
| Nandrolone | 3,828,551 | 7.59 × 10¹² | 24,964.0 | 2.08 × 10⁸ | 33,270.3 | 5.08 × 10⁹ | 90.86 | < 0.0001 | *** | *** | ns |
| Testosterone | 5,681,022 | 5.81 × 10¹³ | 163,261.5 | 3.79 × 10¹⁰ | 74,930.2 | 7.23 × 10⁹ | 44.74 | < 0.0001 | *** | *** | ns |
| 19-norandrosterone | 51,719.9 | 2.16 × 10⁹ | 36,350.9 | 2.15 × 10⁹ | 33,723.5 | 6.80 × 10⁸ | 4.6 | 0.0109 | * | * | ns |
| Androstadienone | 267,066 | 8.98 × 10¹⁰ | 18,530.4 | 2.04 × 10⁸ | 5,987.6 | 3.28 × 10⁷ | 5.23 | 0.0232 | * | * | ns |
| Boldenone | 183,111.9 | 1.51 × 10¹¹ | 111,353.4 | 1.19 × 10¹⁰ | 101,230.9 | 1.60 × 10¹⁰ | 2.67 | 0.071 | ns | ns | ns |
| Androsterone | 5,105,044 | 1.89 × 10¹³ | 4,603,254 | 2.26 × 10¹³ | 3,859,610 | 2.24 × 10¹³ | 1.6 | 0.204 | ns | ns | ns |
| 5α-Androstane-3β,17β-diol | 2,250,145 | 5.36 × 10¹² | 2,502,931 | 6.22 × 10¹² | 1,869,261 | 3.38 × 10¹² | 1.52 | 0.2199 | ns | ns | ns |
| DHEA | 3,736,530 | 5.33 × 10¹⁴ | 1,611,612 | 4.11 × 10¹² | 1,851,250 | 5.94 × 10¹² | 0.65 | 0.5252 | ns | ns | ns |
| Androst-4-ene-3,17-dione | 45,041.1 | 2.55 × 10⁹ | 50,858.5 | 2.62 × 10⁹ | 54,960.7 | 1.17 × 10⁹ | 0.23 | 0.7926 | ns | ns | ns |

Note: SD (Standard deviation) are expressed in scientific notation (e.g., 2.64 × 10¹⁰) due to the extremely large scale of raw intensity peak heights, preserving readability without losing magnitude context.
